# The Effect of Surface Substrate Treatments on the Bonding Strength of Aluminium Inserts with Glass-Reinforced Poly(phenylene) Sulphide

**DOI:** 10.3390/ma15051929

**Published:** 2022-03-04

**Authors:** Ashish Matta, Tomas Sedlacek, Marketa Kadleckova, Anezka Lengalova

**Affiliations:** 1 Department of Polymer Engineering, Faculty of Technology, Tomas Bata University in Zlin, 76001 Zlin, Czech Republic; matta@utb.cz; 2 Centre of Polymer Systems, University Institute, Tomas Bata University in Zlin, 76001 Zlin, Czech Republic; m1_kadleckova@utb.cz (M.K.); lengalova@utb.cz (A.L.); 3 Department of Physics and Materials Engineering, Faculty of Technology, Tomas Bata University in Zlin, 76001 Zlin, Czech Republic; 4 Language Centre, Faculty of Humanities, Tomas Bata University in Zlin, 76001 Zlin, Czech Republic

**Keywords:** polymer–metal bonding, PPS–aluminium bi-component, surface modification

## Abstract

Materials composed of a polymer matrix reinforced with carbon/glass fibres providing lightweight and superior mechanical properties are widely used as structural components for automotive and aerospace applications. However, such parts need to be joined with various metal alloys to obtain better mechanical performance in many structural elements. Many studies have reported enhancements in polymer–metal bonding using adhesives, adhesive/rivet combined joints, and different surface treatments. This study investigated the influences of various surface treatments on the adhesion between glass-reinforced poly(phenylene) sulphide (PPS) and aluminium alloy during the injection over-moulding process. Adhesion strength was evaluated via the shear test. Correlations for the shear strength of the polymer–metal with different metal–substrate treatments were studied. Since the strongest bonding was attained in the treatment with the highest roughness, this value, as it determines the level of micromechanical interlocking of connected materials, seems to be a critical factor affecting the adhesion strength. Three-dimensional (3D) topographic images characterized with a 3D optical microscope indicated that there was a meaningful influence exerted by the interface topologies of the aluminium substrates used for the over-moulding process. The results further indicated that increases in a substrate’s surface energy in connection with atmospheric plasma treatments negatively influence the final level of the bonding mechanism.

## 1. Introduction

Polymer–metal bi-component structures (PMBSs) are expected to be efficient energy-saving parts. They are lightweight with excellent mechanical strength, and they have become prevalent in automobile, aerospace, medical, and electronic applications. Combining the outstanding characteristics of metals and polymer in PMBSs provides high stiffness and stability while significantly reducing the weight of the part [1].

Difficulties involving a lack of adhesion have hindered the development of high-performance thermoplastic–metal bi-component products. The typical joining process employs mechanical joining methods in which plastic material is put through metal holes to form plastic rivets for joining. Another method employs adhesive bonding, in which the metal and the polymer are joined using a suitable adhesive [2,3]. Despite their extensive use in the industry, such techniques have drawbacks, such as stress resulting from riveting, uneven load distributions, and increased structural weight. Usage of adhesives is still challenging due to their low thermal and mechanical characteristics. In this context, a newly designed, ultra-high-temperature-resistant epoxy glue has been employed [4,5].

Another possibility for direct metal–plastic joining is represented by injection over-moulding processes. There are currently three main kinds of polymer–metal hybrid technologies in use in the industry: (a) injection over-moulding, (b) metal over-moulding, and (c) adhesive bonding. In the injection over-moulding process, metal inserts with through-holes are stamped, put in an injection mould, and then over-moulded with a polymer. The thermoplastics are directly injected into the metal surface to achieve a good connection between the metal and plastic components. In the metal over-moulding technique, a metal stamp is placed in an injection mould and coated on the bottom with a thin layer of reinforced polymer. The metal insert’s plastic-coated surface is then welded with an injection-moulded sub-component. In adhesive bonding, a fibre-reinforced polymer is joined to a metal stamp using a thermosetting adhesive. Here, the fatigue strength of the adhesive joints can be improved through the use of rivets. The insertion of close-fitting, adhesively bonded rivets in holes can provide significant improvements to fatigue life [6,7,8].

The surface structure of metals is known to have a substantial impact on metal–polymer bonding strength in general [4,9]. The bonding strength can be improved by changing the surface roughness or modifying the surface chemistry via preparations of the surfaces of metal parts, such as abrasion, etching, and plasma treatment [5,10,11]. To improve the bonding of hybrid joints, laser structuring is used as an alternative to the mechanical blasting process when joining metal with plastics. In these cases, a pulsed laser is focused on a single spot on the material surface, resulting in extremely high local intensities. Furthermore, laser structuring creates different absorption behaviour in the laser radiation at the metal surface, the consequence of which is a variable temperature distribution that can possibly influence the joining result [12]. Moreover, the plastic/metal combination requires specific conditions to reach optimum mechanical performance. Various studies have investigated the joining of polymer–metal bi-components with polymers such as polyether-ether-ketone (PEEK), polypropylene, and thermoplastic polyurethane [8,10,13,14]. There is a broad range of metal–plastic combinations and various possibilities for surface treatments; the present study only focused on evaluating the influence of selected chemical and physical treatments of aluminium inserts, concentrating on the shear bonding strength achieved during injection over-moulding with glass-fibre-reinforced PPS. The main reason for adopting PPS was that there has been very little research on combining PPS with aluminium, and thus there is little information available on combining PPS and aluminium [4]. A glass-reinforced polymer is preferred to increase toughness and strength [6]. PPS is very similar to PEEK but has a lower operating temperature. It is also less expensive; therefore, it could replace PEEK in applications where flexibility is not a key consideration.

## 2. Materials and Methods

Poly(phenylene) sulphide, Fortron^®^ 1140L4, produced by Celanese Corporation (Wilmington, NC, USA) with a density of 1.649 g/cm^3^, melting temperature of 280 ℃, specific heat melt capacity of 1500 J/kg. K, and melt flow index of 28 g/10 min (300 °C/5 kg), filled with 40 wt.% glass fibres, was chosen as a high-performance polymeric material. Before its thermoplastic processing, the material was dried at 130 °C for 3–4 h using a hot air dehumidifying drier. Aluminium alloy AW5754-H111 (Al) with a density of 2.67 g/cm^3^ was used as a lightweight structural material for inserts with a rectangular shape (55 mm × 20 mm × 1 mm).

### 2.1. Surface Pre-Treatment of Al Inserts

**Figure 1:**
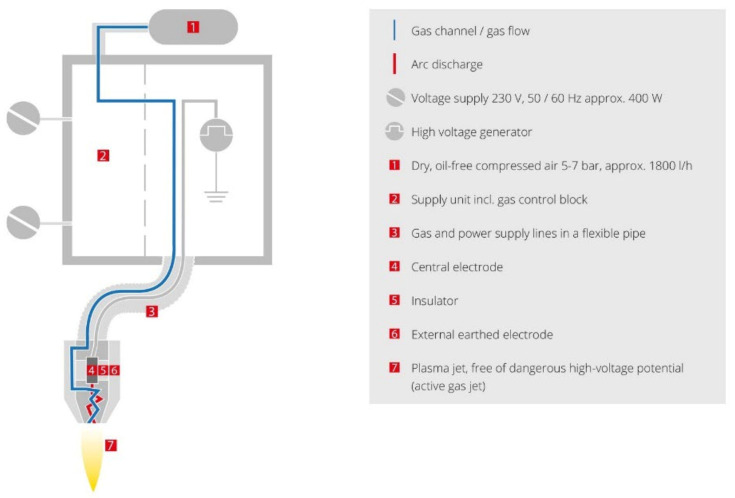
Scheme for the atmospheric pressure plasma system by Diener Electronic [15].

Bonding parts of Al inserts (peripheral parts with dimensions of 10 mm × 20 mm) were cured with suitable surface treatments before the over-moulding to increase the mechanical performance of the adhesion of the materials. Five distinct surface treatments were applied to the inserts. Firstly, the Al inserts were cleaned with acetone. This cleaning procedure was intended as a reference for the following comparison and is presented as the “untreated” specimen in the text below. The different surface treatments were undertaken to better understand the effect of the surface treatment process on the mechanical properties of the final testing specimens. These treatments are referred to as “chemical 1”, “chemical 2”, “atmospheric plasma”, “sandblasting”, and “sandblasting and plasma (S + P) combination”. The details of the surface treatments are provided below:

(a) Chemical 1: Bonding parts of Al inserts were dipped into etching solution (27.5 mL of H_2_SO_4_ (96%) + 7.5 mg of Na_2_Cr_2_O_7_ + 65 mL H₂O). The solution was heated to 65 ℃ for 1–15 min before dipping the inserts. Subsequently, the treated inserts were rinsed with distilled water and dried at the same temperature for 10 min in an oven.
(b) Chemical 2: Bonding parts of Al inserts were treated in the same way as with chemical 1, but NaCl was used instead of Na_2_Cr_2_O_7_.
(c) Plasma treatment: For surface modification of the Al inserts, a Plasma Beam Standard/PC (Diener Electronics, Ebhausen, Germany) was used at ambient temperature and atmospheric pressure. This is a device for cleaning and activating surfaces that has two nozzles with a maximum surface distance of 12 mm. One of the nozzles is presented in Figure 1 [15]. Completely clean and oxide-free surfaces can be obtained, as they are chemically struck by oxygen or air.
(d) The surface energy was measured with contact angles at different distances and different times. The distance (8 mm) and time (20 s) were optimized to obtain the maximum effect from the plasma beam. The distance of 8 mm between the nozzle and the surface of the treated inserts was fixed. The gas used for the plasma treatment was introduced to the Al inserts. The discharge gas (compressed air) was generated using a frequency of 20 kHz and plasma power of 300 W AC, the gas flow rate was 11.2 L/min, and the cooling gas was maintained at 23.7 L/min. Unleaded air was used as a cooling gas.
(e) Sandblasting: An in-depth abrasion treatment was carried out for the substratum using an SBC420 instrument (Reno-Tech s.r.o, Kaznejov, Czech Republic). As an abrasive material, slag (with the composition SiO_2_ 30%, Al_2_O_3_ 40%, CaO 30%) and 120 µm grains were used. The process was performed at a pressure of about 110 kPa and a distance of 8–10 cm from the nozzle for 50 s, at a right angle to the substrate surface [16].
(f) S + P combination: Sandblasting was undertaken in the same way as described above; this was followed by plasma treatment. Inserts were cleaned with acetone after sandblasting and dried before plasma treatment.

### 2.2. Al Insert Surface Morphology Description

Selected methods were used for the evaluation of the effectiveness of the applied surface treatments:

(1) To describe the surface morphologies of the differently treated Al inserts a high-resolution scanning electron microscope (SEM) Phenom Pro X (Waltham, MA, USA) with an electron accelerating voltage of 5 kV was used.
(2) The surface roughness (Ra) of the Al inserts was evaluated with a TR100 surface roughness tester (Time Group Inc, Beijing City, China), and the surface roughness (Sa) was characterized with a 3D optical microscope from 3D images. A Contour GT-K (Bruker, Tucson, AZ, USA) based on white light interferometry was used with a 20× objective lens. The resulting 3D topography maps were processed using Gwyddion 2.55 software (Brno, Czech Republic).
(3) The contact angles of the tested surfaces were analysed using a Surface Energy Evaluation System (SEE System; Advex Instruments, Brno, Czech Republic). The apparatus was used to visualize the drop’s tangent (right and left) and the three-phase points. Each representative contact angle was calculated by averaging at least ten separate readings for every sample (the results are presented in Table 1). Deionized water was used as a testing liquid, and digital images of a 2 µL water droplet on the surface were captured with a charge-coupled device camera system.

The contact angles of two different liquids (water and N-dimethyl formamide) were measured to determine the surface energy using the Owens–Wendt equation (Equation (1)).

(1) $$\cos\theta +1=\frac{2{\left(\gamma_{S}^{D}\gamma_{L}^{D}\right)}^{1/2}}{\gamma_{L}}+\frac{2{\left(\gamma_{S}^{P}\gamma_{L}^{P}\right)}^{1/2}}{\gamma_{L}}$$

The surface tension for both solids and liquids can be obtained as the sum of the dispersion components ($\gamma_{S}^{D}$) and polar components ($\gamma_{S}^{P}$), as given in Equations (2) and (3).

(2) $$\gamma_{S}=\gamma_{S}^{D}+\gamma_{S}^{P}$$

(3) $$\gamma_{L}=\gamma_{L}^{D}+\gamma_{L}^{P}$$

where $\gamma_{S}^{D}$ and $\gamma_{S}^{P}$ are obtained and further substituted in Equation (1).

If at least two liquids are used and the equilibrium contact angle is measured, then Equation (1) can be solved simultaneously to obtain the values of the dispersion and the polar components of the solid sample. They are added to obtain the total surface energy of the sample.

For the calculations, the following values were used for the polar and dispersion components, respectively (in mJ/m^2^): water 51 and 21.8, N-dimethylformamide 13.7 and 17.4.

### 2.3. Preparation of Bi-Component Specimens via Injection Over-Moulding

**Figure 2:**
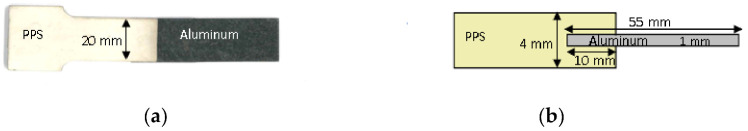
Shear test specimen: (**a**) real picture—top view; (**b**) side-view sketch.

To fabricate the PPS–Al bi-component specimen, the polymer was injected, as presented in Figure 2, using a Mitsubishi180 MEtⅢ (Yokohama, Japan) electric injection moulding machine with a 46 mm diameter screw. For PPS materials in industrial applications, injection temperatures of 300–340 ℃, pressures of 80–130 MPa, and a holding pressure of 80% of the injection pressure are recommended [17]. In our experiment, the process parameters listed in Table 2 were set for injection moulding. Generally, the injection moulding process parameters significantly influence the bonding strength of PPS–Al bi-component parts with the same surface roughness, so the parameters were kept constant to facilitate the description of the effects of the surface treatment. The mould was kept closed for 15 s before injection to heat the Al insert at 120 °C. A cold Al insert would cause polymer melt freezing upon contact, and consequently cause the apprehension of its micron-size roughness features to fail [6].

The shear Testing of over-moulded specimens was carried out with the help of an M350-5CT universal testing machine (Testometric Co. Ltd., Rochdale, UK) equipped with a load cell of 10 kN. All measurements were conducted using a tensile rate of 2 mm/min and a gauge length of 50 mm. For each surface treatment, eight specimens were evaluated for further comparison.

## 3. Results and Discussions

### 3.1. Surface Morphologies of Al Inserts

#### 3.1.1. SEM

**Figure 3:**
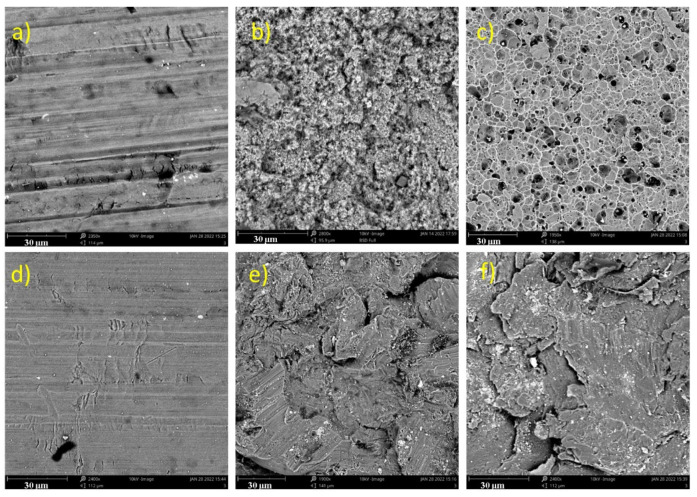
Scanning electron microscopy (SEM) images of the surfaces of the (**a**) untreated, (**b**) chemical 1, (**c**) chemical 2, (**d**) plasma, (**e**) sandblasting, and (**f**) sandblasting + plasma inserts.

The surface microstructures of the Al inserts captured by SEM are presented in Figure 3. While only trivial changes in the surface morphologies could be found for the AL substrates after plasma treatments, the surface roughness of the chemically treated and sandblasted inserts significantly increased. Moreover, visible surface holes and sharp scratches could be detected for the chemical 2 treatment. On the other hand, sandblasted specimens exhibited apparent rough and eroded structure patterns.

The reason that only trivial changes in the surface morphologies could be found after plasma treatment was that this treatment only cleans and soothes the treated surface. No sharp scratches or surface holes were observed after the chemical 1 treatment, in contrast to the chemical 2 treatment, as chemical 2 produced more surface erosion as a result of chemical erosion. On the other hand, sandblasting led to a dramatic increase in the surface roughness, as it is a mechanical treatment undertaken by bombarding the sand particles on the treated surface.

#### 3.1.2. Roughness

In general, the roughness and pore size of a morphology surface crucially affect the adhesion, and they can be influenced by affecting the thermoplastic melt at the level of the metal substrates’ micron-size roughness [7]. A rough topography encourages polymer to flow into metal cavities and increases the overall area at the interface.

**Figure 4:**
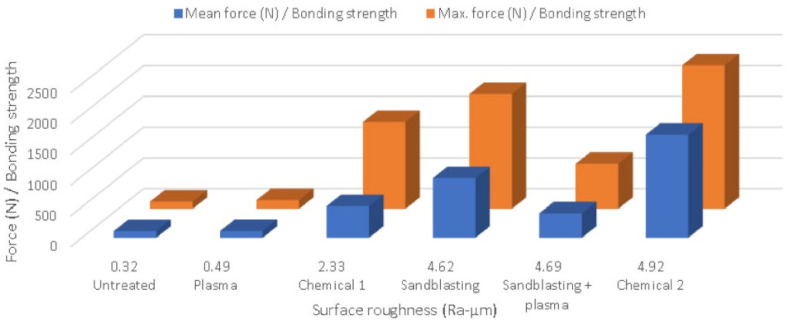
Bonding strength vs. surface roughness.

The highest roughness was obtained for chemical 2 and this treatment had the highest bonding strength as well (see Figure 4, which is ordered by surface roughness). However, the S + P treatment had a lower bonding strength than the chemical 1 and sandblasting treatments, despite having higher roughness. Therefore, it cannot be concluded that a higher roughness means a higher bonding strength.

In general, plasma-treated surfaces usually involve an increase in bonding strength; hence, this was chosen as a preferred method of surface treatment along with sandblasting. However, unlike in a previous study [18], it did not prove to be successful for Al and PPS in our research.

**Figure 5:**
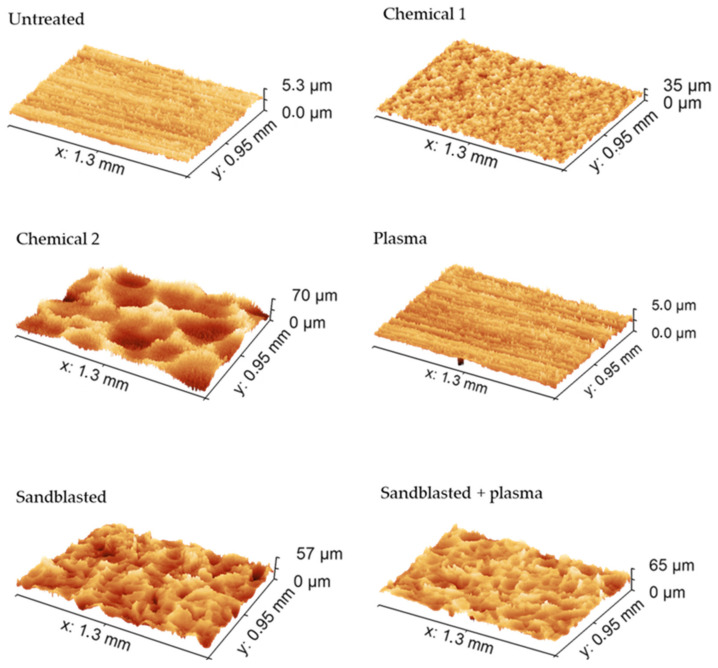
Three-dimensional (3D) images of Al substrate surfaces obtained with a 3D optical microscope.

Figure 5 shows 3D images of the surface-treated Al inserts obtained with a 3D optical microscope. These are in good agreement with the data presented in Table 3.

#### 3.1.3. Contact Angle

Aluminium possesses low surface energy with contact angles of 80°. A contact angle change indicates that the surface has been treated effectively. A reduction of the contact angle indicates an increase in surface energy (Table 1), which in general leads to an increase in bonding strength.

The contact angle measurement results can be seen in Table 1. It is obvious that they were drastically reduced after the chemical and plasma treatments. On the other hand, sandblasting only modestly decreased the contact angle, which was expected since only the topography changed, not the chemical structure.

### 3.2. Bonding Strength of the Bi-Component Specimen

After the surface treatment, all six types of aluminium inserts (eight pieces in each treatment) were used for injection over-moulding. Lap shear strength tests were performed to observe the effect of metal surface treatment on the bond strength between the metal part and the polymer. It should be noted that there were no residues on either the Al insert of the polymer material or the PPS of the metallic substance after the lap shear strength test. Adhesive failure was moreover proved by a comparison of the roughness (Sa) before and after the shear test, as shown in Table 3.

The determined bonding strengths for PPS–Al inserts are presented in Table 4. The mean force was calculated from the average of eight pieces from each treatment, and the maximum force represents the highest value achieved from the eight pieces. As can be seen, as well as having the highest roughness among all the substrates, the chemical 2 treatment also had the highest bonding strength. Nevertheless, it cannot be concluded that the bonding strength is directly proportional to the roughness, as the chemical 1 treatment had a higher bonding strength than the S + P treatment despite the lower roughness.

**Figure 6:**
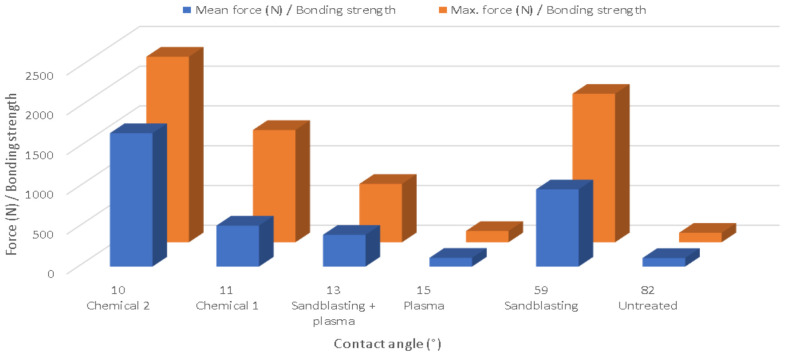
Bonding strength vs. contact angle.

As can be seen in Figure 6 (where the substrates are ordered by contact angle), the plasma treatment obviously reduced the contact angles but did not raise the adhesion or bonding strength much. Moreover, the bonding strength was negatively affected (reduced) when plasma was applied after sandblasting, thus resulting in excellent bonding strength in sandblasted specimens, greater than the S + P samples despite their higher contact angle. This effect could have resulted from the prevention of the PPS melt stream from effectively in-leaking into the created surface knobs due to the increased substrate surface energy.

## 4. Conclusions

This study examined different combinations of mechanical and chemical treatments for aluminium substrates and their effects on the adhesion between metal and poly(phenylene) sulphide. The conclusions can be formulated as follows.

The results indicated that the surface roughness had a remarkable effect on the bonding strength, which was presumably connected with the intrusion of the thermoplastic melt into the metal substrates’ micron-size roughness features. This effect was enhanced by the increased temperature of the metal substrates during the over-moulding process. However, the roughness was not entirely responsible for the good adhesion. Adequate pore sizes and their micro-structuring, determining the active surface of the substrates with the same roughness, also played vital roles in good bonding.

**Figure 7:**
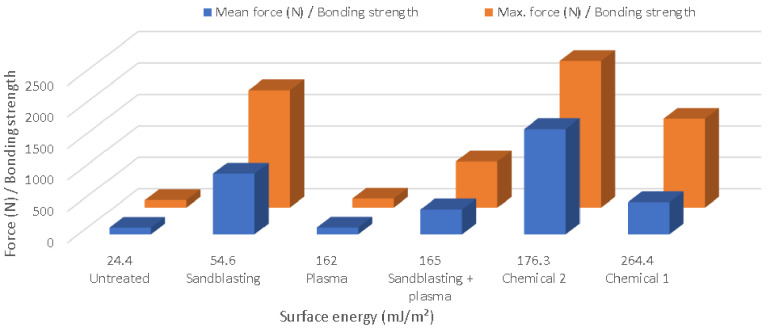
Surface energy vs. bonding strength.

On the other hand, the experiments did not reveal any direct correlation between surface energy and bond strength for this type of polymer/metal connection, as shown by Figure 7. Though the atmospheric plasma treatment increased the surface energy, it did not lead to an improvement in the bonding strength, unlike in previous studies. Instead, its application following sandblasting led to a decline in strength, probably due to the opening of small pores. Therefore, further studies focused on more detailed descriptions of the effect of the roughness topography, in potential combination with plasma treatment, would be helpful in assessing their influence on the bonding strengths of plastics with different polarities.

## Figures and Tables

**Table 1:** Contact angles (deionised water) and surface energies of the treated Al inserts and PPS.

Types of Aluminium Inserts/PPS Surface	Contact Angle (°)	Surface Energy (mJ/m^2^)	Deviation Surface Energy
Untreated	82	24.4	± 1.5
Chemical 1	11	264.4	± 12.4
Chemical 2	10	176.3	± 6.2
Plasma	15	162.0	± 5.5
Sandblasting	59	54.6	± 3.1
Sandblasting + plasma	13	165.0	± 4.8
PPS	99	32.3	± 1.2

**Table 2:** Injection moulding process parameters.

Injection Speed (mm/s)	130
Injection pressure (MPa)	60
Cooling temperature under the hopper (℃)	40–50
Zones 1, 2, 3, and 4 temperatures (℃)	290, 310, 330, 330
Nozzle temperature (℃)	310
Holding pressure (MPa)	45
Holding time (s)	7
Cooling time (s)	15
Mould temperature (℃)	120

**Table 3:** Surface roughness (Sa; µm) measured by optical profilometry.

	Untreated	Chemical 1	Chemical 2	Plasma	Sandblasting	Sandblasting + Plasma
Roughness (Sa; µm) before shear test	0.5 ± 0.1	3.1 ± 0.6	7.9 ± 1.4	0.90 ± 0.1	6.5 ± 0.8	6.7 ± 0.5
Roughness (Sa; µm) after shear test	0.4 ± 0.2	3.1 ± 0.5	8.0 ± 1.2	0.5 ± 0.1	6.5 ± 0.7	6.2 ± 0.2

**Table 4:** The bonding strength and surface roughness of samples fabricated with different surface preparations.

Types of Aluminium Inserts	Surface Roughness (Ra; µm)	Deviation of Surface Roughness (Ra; µm)	Bonding Strength/Max. Force (N)	Bonding Strength/Mean Force (N)
Untreated	0.32	± 0.06	119	108
Chemical 1	2.33	± 0.83	1411	515
Chemical 2	4.92	± 1.86	2332	1676
Plasma	0.49	± 0.09	142	110
Sandblasting	4.62	± 1.49	1866	970
Sandblasting + plasma	4.69	± 1.27	734	399

## Data Availability

Not applicable.

## References

[1] Li X., Liu F., Gong N., Yang C., Wang B. (2018). Surface Topography Induced High Injection Joining Strength of Polymer-Metal Composite and Fracture Mechanism. Compos. Struct..

[2] Martinsen K., Hu S.J., Carlson B.E. (2015). Joining of Dissimilar Materials. CIRP Ann. Manuf. Technol..

[3] Mandolfino C., Lertora E., Gambaro C. (2013). Effect of Surface Pretreatment on the Performance of Adhesive-Bonded Joints. Key Engineering Materials.

[4] Xu Z., Li N. (2020). Effects of Surface Microstructure and Molding Parameters on Injection Bonding Strength of Polyphenylenesulphide-Aluminum Alloy. Mater. Express.

[5] Iqbal H.M.S., Bhowmik S., Poulis J.A., Benedictus R. (2010). Effect of Plasma Treatment and Electron Beam Radiations on the Strength of Nanofilled Adhesive-Bonded Joints. Polym. Eng. Sci..

[6] Lucchetta G., Marinello F., Bariani P.F. (2011). Aluminum Sheet Surface Roughness Correlation with Adhesion in Polymer Metal Hybrid Overmolding. CIRP Ann.—Manuf. Technol..

[7] Grujicic M., Sellappan V., Omar M.A., Seyr N., Obieglo A., Erdmann M., Holzleitner J. (2008). An Overview of the Polymer-to-Metal Direct-Adhesion Hybrid Technologies for Load-Bearing Automotive Components. J. Mater. Process. Technol..

[8] Ochoa-Putman C., Vaidya U.K. (2011). Mechanisms of Interfacial Adhesion in Metal-Polymer Composites—Effect of Chemical Treatment. Compos. Part A Appl. Sci. Manuf..

[9] Shahid M., Hashim S.A. (2002). Effect of Surface Roughness on the Strength of Cleavage Joints.

[10] Iqbal H.M.S., Bhowmik S., Benedictus R. (2010). Surface Modification of High Performance Polymers by Atmospheric Pressure Plasma and Failure Mechanism of Adhesive Bonded Joints. Int. J. Adhes. Adhes..

[11] Kadlečková M., Minařík A., Smolka P., Mráček A., Wrzecionko E., Novák L., Musilová L., Gajdošík R. (2018). Preparation of Textured Surfaces on Aluminum-Alloy Substrates. Materials.

[12] Heckert A., Zaeh M.F. (2014). Laser Surface Pre-Treatment of Aluminium for Hybrid Joints with Glass Fibre Reinforced Thermoplastics. Phys. Procedia.

[13] Moritz J., Götze P., Schiefer T., Stepien L., Klotzbach A., Standfuß J., López E., Brückner F., Leyens C. (2021). Additive Manufacturing of Titanium with Different Surface Structures for Adhesive Bonding and Thermal Direct Joining with Fiber-Reinforced Polyether-Ether-Ketone (PEEK) for Lightweight Design Applications. Metals.

[14] Gardiner G. (2015). Overmolding Expands PEEK’s Range in Composites. CompositesWorld.

[15] (2020). Handbook of Plasma Surface Technnology—Plasma Technology.

[16] Miturska-Barańska I., Rudawska A., Doluk E. (2021). The Influence of Sandblasting Process Parameters of Aerospace Aluminium Alloy Sheets on Adhesive Joints Strength. Materials.

[17] Wurzbacher S., Gach S., Reisgen U., Hopmann C. (2021). Joining of Plastic-Metal Hybrid Components by Overmoulding of Specially Designed Form-Closure Elements (Fügen von Kunststoff-Metall-Hybridbauteilen Durch Das Hinterspritzen Gezielt Aufgebauter Formschlusselemente). Mater. Werkst..

[18] Leahy W., Barron V., Buggy M., Young T., Mas A., Schue F., McCabe T., Bridge M. (2001). Plasma Surface Treatment of Aerospace Materials for Enhanced Adhesive Bonding. J. Adhes..

